# Antimicrobial Effects of Equine Platelet Lysate

**DOI:** 10.3389/fvets.2021.703414

**Published:** 2021-08-19

**Authors:** Julie Gordon, Sonsiray Álvarez-Narváez, John F. Peroni

**Affiliations:** ^1^Department of Large Animal Medicine, Veterinary Medical Center, College of Veterinary Medicine, University of Georgia, Athens, GA, United States; ^2^Athens Veterinary Diagnostic Laboratory, Department of Infectious Diseases, College of Veterinary Medicine, University of Georgia, Athens, GA, United States

**Keywords:** equine, antibiotic alternative, broad-spectrum antimicrobial, *E. faecalis*, *P. aeruginosa*, *E. coli*, *S. aureus*, platelet lysate

## Abstract

The development of antimicrobial resistant bacteria and the lack of novel antibiotic strategies to combat those bacteria is an ever-present problem in both veterinary and human medicine. The goal of this study is to evaluate platelet lysate (PL) as a biological alternative antimicrobial product. Platelet lysate is an acellular platelet-derived product rich in growth factors and cytokines that is manufactured via plateletpheresis and pooled from donor horses. In the current study, we sought to define the antimicrobial properties of PL on select gram-positive and gram-negative bacteria. Results from an end-point *in vitro* assay showed that PL did not support bacterial growth, and in fact significantly reduced bacterial content compared to normal growth media. An *in vitro* assay was then utilized to further determine the effects on bacterial growth dynamics and showed that all strains exhibited a slower growth rate and lower yield in the presence of PL. The specific effects of PL were unique for each bacterial strain: *E. coli* and *P. aeruginosa* growth was affected in a concentration-dependent manner, such that higher amounts of PL had a greater effect, while this was not true for *S. aureus* or *E. faecalis*. Furthermore, the onset of exponential growth was delayed for *E. coli* and *P. aeruginosa* in the presence of PL, which has significant clinical implications for developing a dosing schedule. In conclusion, our findings demonstrate the potential value of PL as a broad-spectrum antimicrobial that would offer an alternative to traditional antibiotics for the treatment of bacterial infection in equine species.

## Introduction

Biological products derived from platelets have been extensively studied as potential therapies because they are a rich source of bioactive elements including growth factors, which are thought to promote healing of injured tissues (1–5). Once platelets are collected and concentrated, several techniques can be employed to promote the release of these growth and chemotactic factors present in their alpha granules. These factors are captured in what is known as a platelet releasate or platelet lysate (PL), which can be further refined in the lab to remove cellular debris resulting in an acellular product. In horses, most platelet-derived products currently used for therapeutic purposes are autologous, generated on an as-needed basis from the same patient in which they will be used. In contrast, we have established a safe large scale plateletpheresis process to produce PL from multiple donor animals (6). After pheresis, PL is manufactured in the laboratory and pooled from a minimum of three donors to be then stored until required. We have shown that pooled PL is more effective at suppressing cell-mediated inflammation than PL obtained from individual horses (6), possibly because it contains a more consistent balance of effector proteins.

In addition to being well-known regulators of thrombosis and inflammation, there is mounting evidence to suggest that platelets also function in the host response to infection (7). Platelets kill bacteria by producing oxygen metabolites such as superoxide, hydrogen peroxide, and hydroxyl free radicals (8). Moreover, platelets participate in antibody-dependent cell cytotoxicity against microbial pathogens (9). These effects are thought to be mediated either by a direct interaction between platelets and bacteria or, perhaps more interestingly, via peptides released by activated platelets (8, 10–12).

Based on this evidence, we have begun to explore the antimicrobial effects of PL, centering this effort around two overarching concerns. First, bacterial infection in equine species is a serious problem, leading to prolonged, often unsuccessful treatments that heavily burden horse owners both emotionally and financially. Bacterial species commonly responsible for equine infections include *Escherichia coli, Streptococcus* spp, *Staphylococcus* spp, and *Pseudomonas* spp, among others (13, 14). Secondly, discovering effective substitutes for traditional antibiotics is an urgent medical need. In fact, the rapid rise of antibiotic resistance in bacterial pathogens is a paramount global health crisis that has considerable implications for both human and animal health. The prolonged and indiscriminate use of traditional antibiotics is one of many factors contributing to the development of resistant bacteria, further increasing the pressure to develop alternative antimicrobial strategies (15, 16). These concerns prompted the present study based on the hypothesis that PL will inhibit bacterial growth regardless of gram-positive or gram-negative cell wall characterization. Our objectives were, therefore, to employ specific microbiology assays to define the antimicrobial properties of PL on select gram-positive and gram-negative bacteria.

## Materials and Methods

### PL Manufacture

Equine platelet lysate was manufactured via plateletpheresis performed in three mix-breed, healthy adult horses (ages 4 to 14) belonging to the University of Georgia research and teaching herd (IACUC approval #A2018 01-013). One liter of platelet concentrate was obtained from each horse and was subjected to two freeze-thaw cycles to disrupt the platelets and release their contents (6). The product was then centrifuged three times, filtered to remove cell debris, and pooled. The final product was aliquoted into 50 ml tubes and stored at −80°C until ready for use in this study.

### Bacterial Strains and Culture Conditions

The gram-positive bacteria *Staphylococcus aureus subsp. aureus* (ATCC^®^ 49230™) and *Enterococcus faecalis* (ATCC^®^ 29212™) and the gram-negative bacteria *Escherichia coli* (ATCC^®^ 25922™) and *Pseudomonas aeruginosa* (clinical isolate; kind gift from M. Hondalus, UGA) were investigated in this study. All strains were cultured in BBL Brain Heart Infusion broth (BHI, Becton Dickinson, Sparks, MD) at 37°C with shaking (200 rpm). Solid growth media was prepared using Difco Brain Heart Infusion agar (Becton Dickinson).

### *In vitro* Growth Assay

For each strain, several colonies from a fresh pure culture were grown overnight in BHI at 37°C with shaking (200 rpm). An appropriate volume of the bacteria solution was used to measure the optical density at 600 nm (OD_600_) using a BioRad SmartSpec Plus spectrophotometer. OD_600_ was adjusted to 0.02 (corresponding to ~10^7^ CFU/ml). Starting cultures were plated onto BHI plates to determine bacterial content before treatment. The required number of 1 ml aliquots were removed from each and centrifuged at 3,000 rpm for 10 min to pellet the bacteria. Pellets were washed with PBS to remove residual media, then 1 ml of the appropriate treatment (100% BHI or 100% PL) was added. All treatments were performed in triplicate. After overnight incubation at 37°C with shaking (200 rpm), cultures were serially diluted in PBS and plated onto BHI plates. Colonies were counted the following day to determine the number of bacteria present at the end of the treatment period (CFU/ml). Data are presented as fold-change in bacterial number over the 24-h period.

### Growth Curve Assay

Bacteria were grown overnight as described above and the OD_600_ was adjusted to 0.02 (corresponding to approximately 10^7^ CFU/ml). The required number of 1 m1 aliquots was removed from each and centrifuged at 3,000 rpm for 10 min to pellet the bacteria. Pellets were washed with PBS then 500 μl of the appropriate treatment (100%, 80%, 60%, 40%, 20% PL or PBS in BHI as appropriate) was added. Tube contents were transferred in duplicate to a 48-well plate, which was placed into an automated plate reader (Synergy HT, BioTek) and allowed to incubate for 16 h at 37°C, 200 rpm.

OD_600_ (corresponding to the number of bacteria in each well) was measured every 10 min. Blank no-bacteria controls were included for all treatments and values were subtracted from OD_600_ readings. Growth curve data were processed using statistical software “R.” Data were further analyzed using Gen5 3.00 Software (BioTek) and bacterial fitness was measured by determining the maximum bacterial yield (max OD_600_), average growth rate (mean V), and lag time. Growth rate was measured across 2-h intervals for the full 16-h period.

### Statistical Analysis

Data from *in vitro* growth experiments were processed using Gen5 3.00 Software (BioTek). Experimental conditions were set up in duplicate, and averaged to give a value for each condition in each independent experiment. All statistical analysis was performed using GraphPad Prism software. Values were subsequently analyzed to check for significant differences using an unpaired *t*-test (*in vitro* growth assay) or ordinary one-way ANOVA followed by Tukey's multiple comparisons test (growth curve assay). Data are presented as mean ± SD of three independent experiments. For all analyses, *P* < 0.05 was considered significant.

## Results

### PL Does Not Support Bacterial Growth *in vitro*

**Figure 1:**
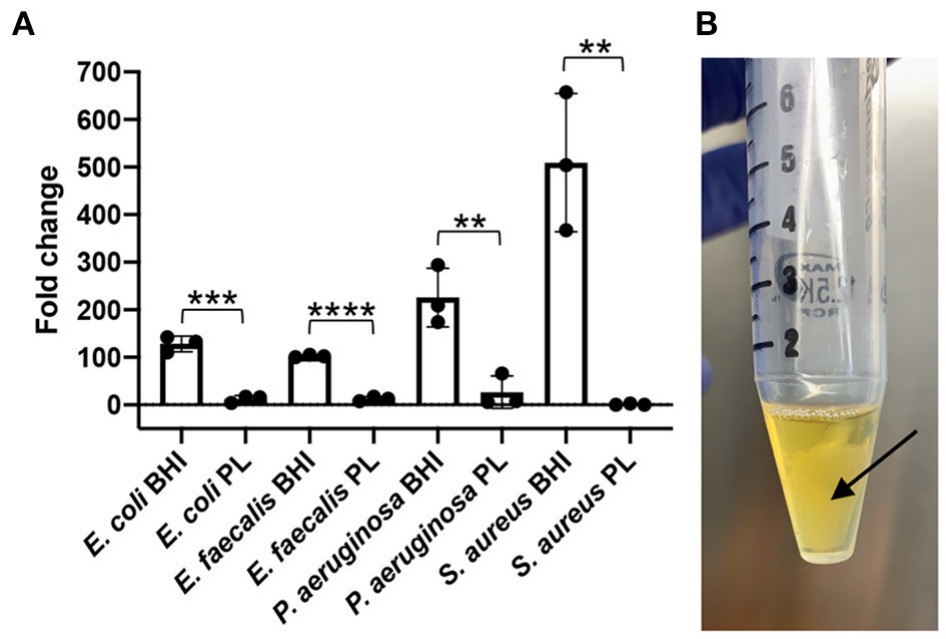
Effect of PL on bacterial growth after 24 h. *E. coli, S. aureus, P. aeruginosa*, or *E. faecalis* were grown in the presence of 100% PL or BHI for 24 h, then CFU/ml was determined after 24 h and compared to starting counts to obtain fold change **(A)**. *S. aureus* formed a floating aggregate after 24 h in PL [**(B)**, arrow). **P = 0.0037 (*S. aureus*) or 0.0081 (*P. aeruginosa*) compared to BHI; ***P = 0.0004 compared to BHI; ****P < 0.0001 compared to BHI. Data represent mean ± SD of three independent experiments. PL, platelet lysate. CFU, colony forming units.

A preliminary end-point *in vitro* assay designed to expose *S. aureus, E. coli, P. aeruginosa* and *E. faecalis* to 100% PL, showed that bacterial content was significantly reduced in the presence of PL compared to control BHI growth media (Figure 1A). Over the 24-h treatment period, bacterial content for *E. coli* increased 128-fold in BHI, compared to only 12-fold in PL (*P* = 0.0004). Similarly, *E. faecalis* increased 102-fold in BHI and only 12-fold in PL (*P* < 0.0001), and *P. aeruginosa* bacterial content increased 226-fold in BHI and 26-fold in PL (*P* = 0.0081). The findings for *S. aureus* could not be interpreted because, rather than remaining in a homogenous solution as was seen in BHI, *S. aureus* coalesced in a free-floating aggregate within a few hours of PL culture (Figure 1B). The aggregate could not be mechanically disrupted to retrieve bacteria for serial dilutions and plating, however, direct plating onto BHI agar resulted in bacterial colonies that were too dense to count (data not shown), confirming that most of the bacteria were alive and had organized into the aggregate.

**Figure 2:**
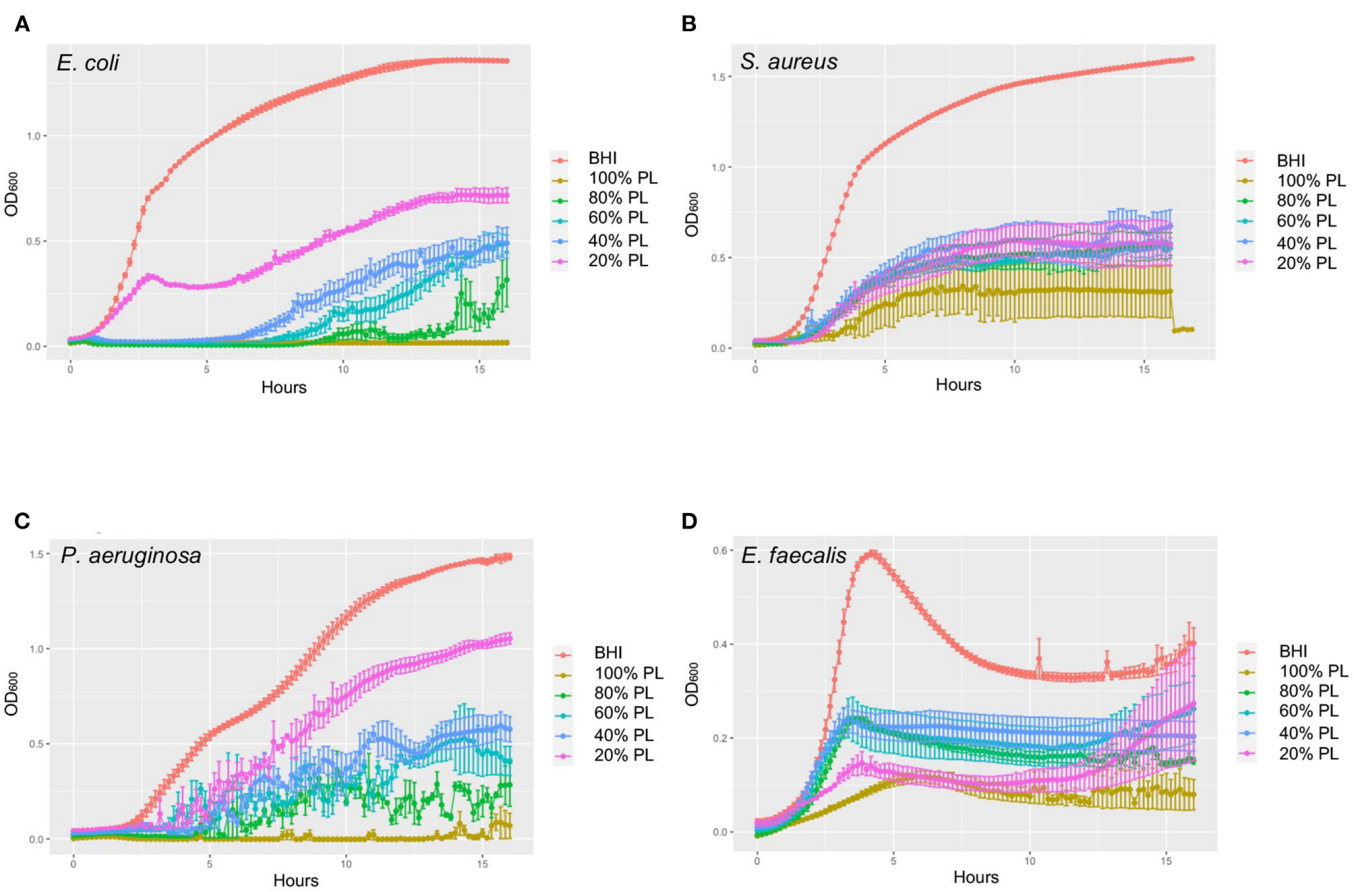
Growth curves showing dynamic changes in growth of *E. coli* **(A)**, *S. aureus* **(B)**, *P. aeruginosa* **(C)**, and *E. faecalis* **(D)** in response to different concentrations of PL compared to BHI. OD_600_ was measured every 10 min for 16 h in an automated plate reader. Data represent mean ± SD of three independent experiments. PL, platelet lysate.

Encouraged by this preliminary experiment, we chose to then employ a growth curve assay designed to more accurately monitor kinetic changes in bacterial growth over a 16-h period. The results of these experiments are presented in Figure 2, showing growth curves from three independent experiments for *E. coli, S. aureus, P. aeruginosa*, and *E. faecalis*. In a parallel set of experiments, we controlled for any possible effects of PL being related to reduced nutritional support, by substituting PL with the same amount of PBS (Supplementary Figure 1). Because the specific effects of PL were unique for each bacterial strain, we chose to report them separately.

### PL Delays the Exponential Growth of *E. coli* in a Concentration-Dependent Manner

**Figure 3:**
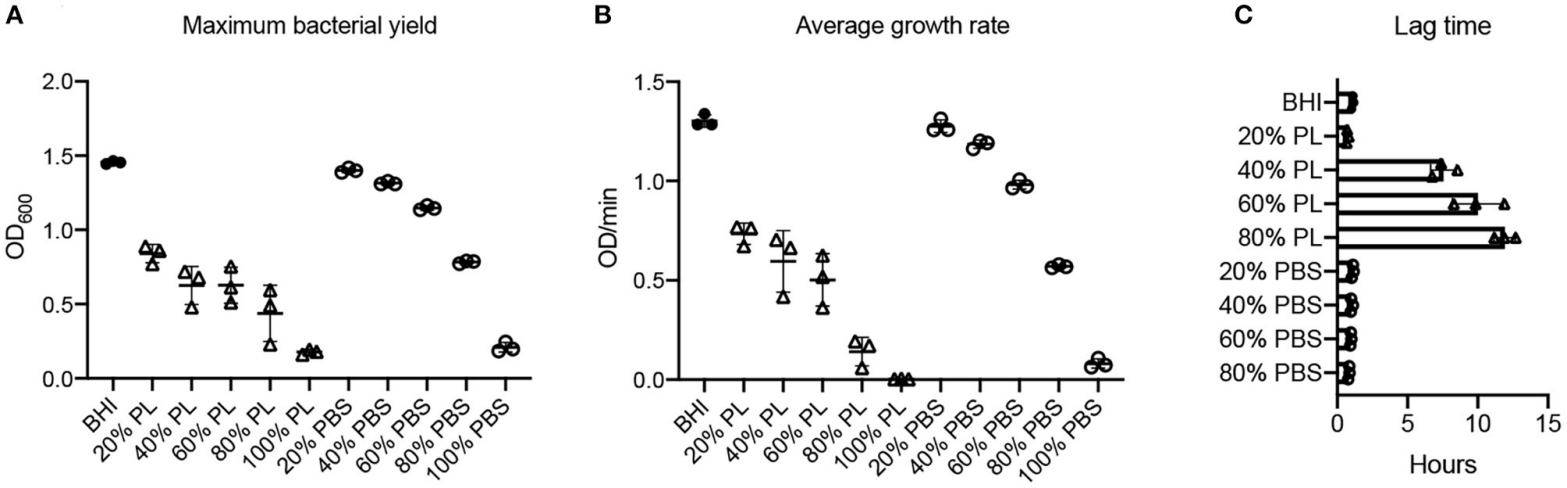
Effect of PL on *E. coli* growth dynamics. Maximum bacterial yield (OD_600_) after treatment with different PL (Δ) or PBS (o) concentrations for 16 h **(A)**. Average growth rate (OD/min) after treatment with different PL (Δ) or PBS (o) concentrations for 16 h **(B)**. Lag time (h) after treatment with different PL (Δ) or PBS (o) concentrations for 16 h **(C)**. Data represent mean ± SD of three independent experiments. PL, platelet lysate. PBS, phosphate buffered saline.

*E. coli* growth was monitored over a 16-h period in the presence of different concentrations of PL (Figure 3). The max OD_600_ data revealed a good correlation with PL concentration, where the maximum bacterial yield decreased as the amount of PL increased (Figure 3A). The max OD_600_ value for bacteria growing in BHI was 1.45, but only 0.84, 0.63, 0.63, 0.44, and 0.18 in 20, 40, 60, 80, and 100% PL, respectively (*P* < 0.0001). Bacterial yield was also affected in the PBS controls (Figure 3A), suggesting that a reduction in BHI may have a direct effect on growth. However, these effects were much less than with PL, and differences from the BHI control were not significant until PBS concentrations reached 60%. The max OD_600_ value fell from 1.45 in BHI to 1.15 in 60% PBS (*P* = 0.0051), 0.78 in 80% PBS (*P* < 0.0001), and 0.21 in 100% PBS (*P* < 0.0001). These values were still significantly higher than the equivalent PL concentration (1.15 in 60% PBS vs. 0.63 in 60% PL *P* < 0.0001, and 0.78 in 80% PBS vs. 0.44 in 80% PL *P* = 0.0013), suggesting that PL is having a direct effect on *E. coli* growth.

Consistent with these data, bacterial growth rate was also affected in a concentration-dependent manner. The average growth rate (mean V) of *E. coli* in BHI was 1.30 OD/min over the 16-h period. This was reduced to 0.73 OD/min with 20% PL, and further to 0.60, 0.50, 0.14, and 0 OD/min with 40, 60, 80, and 100% PL, respectively (Figure 3B). PBS treatment also had an effect on growth rate, although less than with PL (Figure 3B). There was no significant difference in average growth rates between 20 and 40% PBS and BHI. With 60% PBS, the mean growth rate fell to 0.98 OD/min, and was further reduced to 0.57 OD/min in 80% PBS, and 0.08 in 100% PBS. The values for 60 and 80% PBS were significantly higher than the equivalent PL concentration (0.98 OD/min in 60% PBS vs. 0.50 OD/min in 60% PL, and 0.57 OD/min in 80% PBS vs. 0.14 OD/min in 80% PL *P* < 0.0001). Lag time is defined as the period of little or no cell division before the initiation of exponential growth. For *E. coli* in BHI or 20% PL the lag time was around 1 h, but was increased to 8 h, 10 h, and 12 h in 40, 60, and 80% PL, respectively (Figures 2A, 3C). This growth delay was not seen with any of the PBS concentrations, where lag time was consistently around 1 h (Figure 3C, Supplementary Figure 1A), indicating that the delayed growth is a direct effect of PL and not reduced nutritional support.

### PL Affects *S. aureus* Growth and Is Not Concentration-Dependent

**Figure 4:**
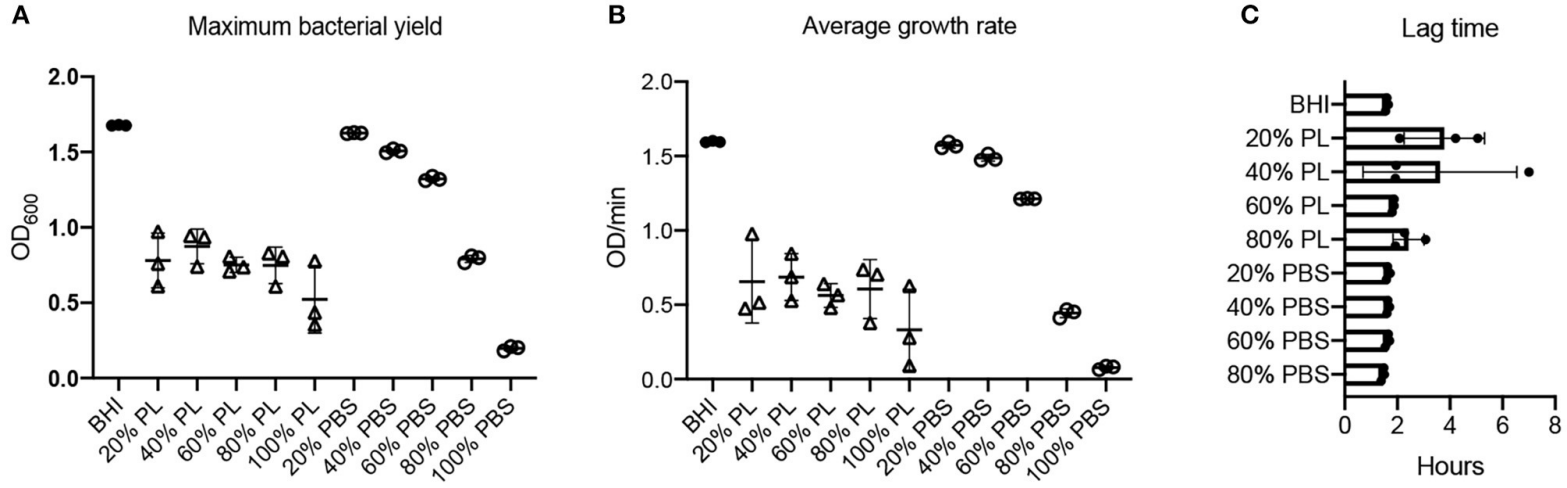
Effect of PL on *S. aureus* growth dynamics. Maximum bacterial yield (OD_600_) after treatment with different PL (Δ) or PBS (o) concentrations for 16 h **(A)**. Average growth rate (OD/min) after treatment with different PL (Δ) or PBS (o) concentrations for 16 h **(B)**. Lag time (h) after treatment with different PL (Δ) or PBS (o) concentrations for 16 h **(C)**. Data represent mean ± SD of three independent experiments. PL, platelet lysate. PBS, phosphate buffered saline.

The effect of PL on *S. aureus* growth dynamics was not concentration-dependent: bacterial yield and growth rate were equally affected in the presence of 20, 40, 60, and 80% PL (Figure 4). The max OD_600_ value for *S. aureus* in BHI was 1.68, and was reduced to 0.78, 0.87, 0.75, 0.75, and 0.52 in 20, 40, 60, 80, and 100% PL, respectively (Figure 4A; *P* < 0.0001). There was no significant difference in max OD_600_ values between 20, 40, 60, and 80% PL, therefore the effect on bacterial yield did not depend on PL concentration. Bacterial yield was also affected by PBS treatment, however, the effect was less than with PL (Figure 4A). There was no significant difference in max OD_600_ values between the BHI control and 20 or 40% PBS. The max OD_600_ value for *S. aureus* fell from 1.68 in BHI to 1.32 in 60% PBS (*P* = 0.0116), to 0.79 in 80% PBS (*P* < 0.0001), and to 0.20 in 100% PBS (*P* < 0.0001). The value for 60% PBS was significantly higher than the equivalent PL concentration (1.32 in 60% PBS vs. 0.75 in 60% PL *P* < 0.0001), but the values for 80% were not significantly different (0.79 in 80% PBS vs. 0.75 in 80% PL), suggesting that there may be some effect of reduced nutrition at higher PL concentrations.

A similar trend was observed for the average growth rate over the 16-h period: the mean V value for *S. aureus* in BHI was 1.60 OD/min and was reduced to 0.66, 0.69, 0.56, 0.61, and 0.33 OD/min in 20, 40, 60, 80, and 100% PL, respectively (Figure 4B; *P* < 0.0001). However, the values for 20, 40, 60, and 80% PL were not significantly different from each other, indicating that the effect was not related to PL concentration. The PBS treatments did show a concentration-dependent effect on the average growth rate for *S. aureus* (Figure 4B). The mean V values for 20, 40, 60, 80, and 100% PBS were 1.57, 1.49, 1.21, 0.44, and 0.08 OD/min, respectively, compared to 1.60 OD/min in BHI. Only 80 and 100% PBS were significantly different from BHI (*P* < 0.0001), but, more importantly, 20, 40, and 60% PBS were significantly higher than the equivalent PL concentration (*P* < 0.0001 for 20 and 40%, *P* = 0.0005 for 60%), indicating that the effects on growth were not solely due to nutrient deprivation. The lag time was similar for all treatments, consistently around 2–4 h (Figures 2B, 4C, Supplementary Figure 1B), indicating no effect on the onset of exponential growth.

### PL Delays the Exponential Growth of *P. aeruginosa* in a Concentration-Dependent Manner

**Figure 5:**
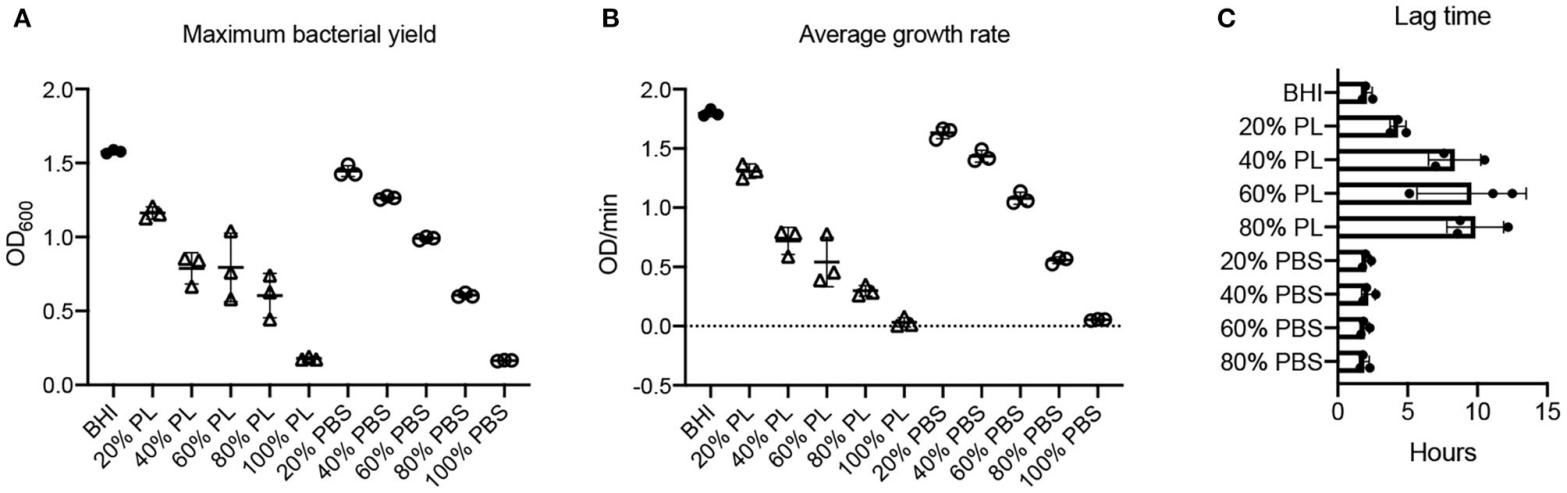
Effect of PL on *P. aeruginosa* growth dynamics. Maximum bacterial yield (OD_600_) after treatment with different PL (Δ) or PBS (o) concentrations for 16 h **(A)**. Average growth rate (OD/min) after treatment with different PL (Δ) or PBS (o) concentrations for 16 h **(B)**. Lag time (h) after treatment with different PL (Δ) or PBS (o) concentrations for 16 h **(C)**. Data represent mean ± SD of three independent experiments. PL, platelet lysate. PBS, phosphate buffered saline.

Bacterial yield and growth rate for *P. aeruginosa* were affected by PL in a concentration-dependent manner (Figure 5). The max OD_600_ value was 1.58 in BHI. This was reduced to 1.16 in 20% PL (*P* = 0.006), and further to 0.87, 0.79, 0.65, and 0.31 in 40, 60, 80, and 100% PL, respectively (*P* < 0.0001), indicating that bacterial yield decreased as the percentage of PL increased (Figure 5A). Bacterial content was also reduced in the PBS treatment groups compared to BHI, although this difference was significant only in the 40% (1.26 vs. 1.44 in BHI *P* = 0.0128), 60% (0.99 vs. 1.44 in BHI *P* < 0.0001), 80% (0.61 vs. 1.44 in BHI *P* < 0.0001), and 100% (0.17 vs. 1.44 in BHI *P* < 0.0001) PBS groups (Figure 5A). Note that the max OD_600_ values for 20 and 40% PBS were significantly higher than for the equivalent PL concentrations (1.45 vs. 1.16 for 20% *P* = 0.00302, and 1.27 vs. 0.87 for 40% *P* < 0.0001), while this was not true for 60, 80, and 100%. This means that for concentrations of 60% and above we cannot distinguish between an effect of PL and reduced BHI.

The average growth rate for *P. aeruginosa* over 16 h was also affected in a concentration-dependent manner (Figure 5B). Mean V values fell from 1.80 OD/min in BHI to 1.31, 0.72, 0.54, 0.30, and 0.03 OD/min in 20, 40, 60, 80, and 100% PL, respectively (*P* < 0.0001). As reported for bacterial yield, growth rate was significantly affected in the 40% (1.43 OD/min), 60% (1.08 OD/min), 80% (0.56 OD/min), and 100% (0.05 OD/min) PBS groups compared to BHI (*P* = 0.0007 for 40%, *P* < 0.0001 for others) (Figure 5B). With the exception of 100%, the effect of PBS vs. PL on growth rate was significantly different at all equivalent concentrations (*P* = 0.028 for 20%, *P* < 0.0001 for 40 and 60%, *P* = 0.0261 for 80%). Lag time for *P. aeruginosa* in BHI was around 2 h, meaning that bacterial cell division and exponential growth did not start until this time (Figures 2C, 5C). For 20% PL treatment this lag period increased to 4 h, and further increased to 9–10 h in 40, 60, and 80% PL (Figures 2C, 5C), indicating a delay in the onset of exponential growth in the presence of PL. Again, this was not seen with the PBS treatments (Figure 5C, Supplementary Figure 1C), and is therefore likely to be a true effect of the PL rather than reduced nutrition.

### PL Affects *E. faecalis* Growth and Is Not Concentration-Dependent

**Figure 6:**
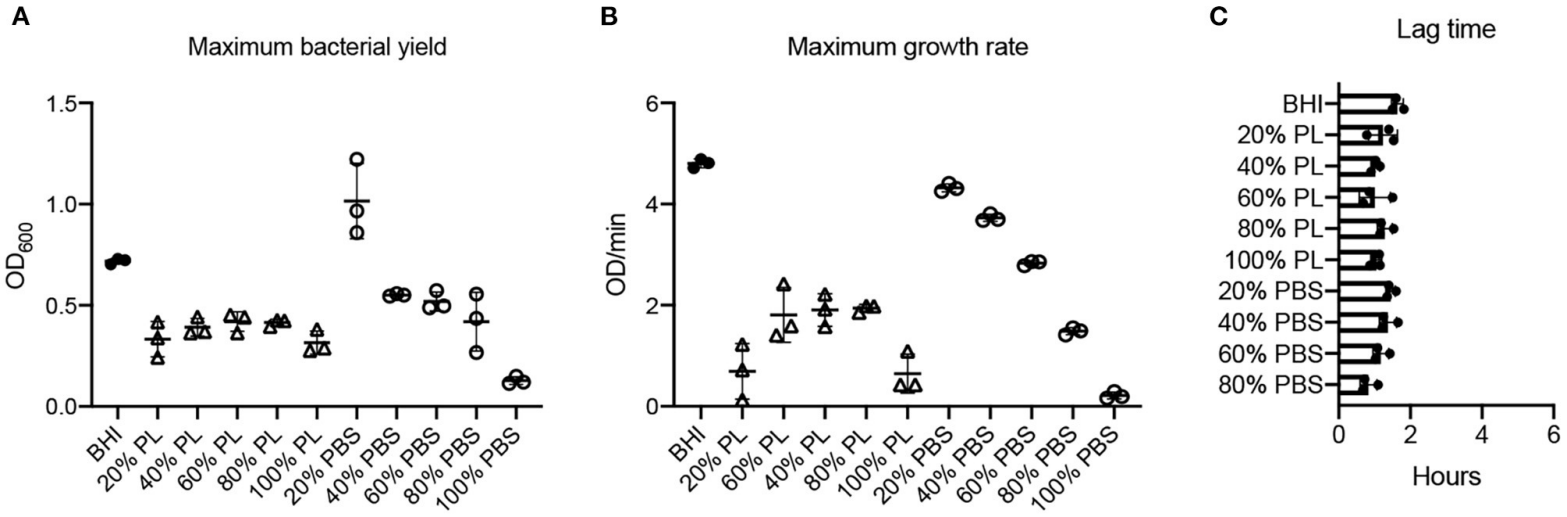
Effect of PL on *E. faecalis* growth dynamics. Maximum bacterial yield (OD_600_) after treatment with different PL (Δ) or PBS (o) concentrations for 16 h **(A)**. Maximum growth rate (OD/min) after treatment with different PL (Δ) or PBS (o) concentrations for 16 h **(B)**. Lag time (h) after treatment with different PL (Δ) or PBS (o) concentrations for 16 h **(C)**. Data represent mean ± SD of three independent experiments. PL, platelet lysate. PBS, phosphate buffered saline.

*E. faecalis* bacterial yield was reduced at all PL concentrations tested (Figure 6A). The max OD_600_ value for *E. faecalis* in BHI was 0.72, which was reduced significantly to 0.33, 0.39, 0.42, 0.42, and 0.32 in 20, 40, 60, 80, and 100% PL, respectively (*P* = 0.0004, 0.0028, 0.0071, 0.0062, 0.0002, respectively). Furthermore, there was no significant difference in max OD_600_ values between any of the PL concentrations, including 100%, indicating that the bacterial yield for *E. faecalis* does not exhibit the same concentration response to PL as some of the other strains in this study. The maximum bacterial yield for PBS treatments showed a concentration response, changing from 0.72 in BHI to 1.02, 0.55, 0.52, 0.42, and 0.13 in 20, 40, 60, 80, and 100% PBS, respectively (Figure 6A).

Values for average growth rate could not be obtained for *E. faecalis* due to the shape of the growth curve in BHI (Figure 2D). However, we were able to derive maximum growth rates from the data and found that they were significantly lower for all PL treatments than for BHI (0.69, 1.91, 1.81, 1.94, and 0.65 OD/min in 20, 40, 60, 80, and 100% PL, respectively, compared to 4.80 OD/min in BHI *P* < 0.0001) (Figure 6B). Growth rate was significantly less with 100% PL than for all other concentrations, except 20% (*P* = 0.0005 for 80%, *P* = 0.002 for 60%, *P* = 0.0007 for 40%), and 40, 60, and 80% PL were not significantly different from each other, indicating that PL does not have a concentration-dependent effect on *E. faecalis* growth. There was a direct correlation between BHI nutritional support and growth rate, as shown by the PBS controls: max V values decreased from 4.80 OD/min in BHI to 4.32, 3.73, 2.81, 1.49, and 0.22 OD/min in 20, 40, 60, 80, and 100% PBS, respectively (Figure 6B). At all concentrations except 20%, the growth rate was significantly different from BHI (*P* = 0.0045 for 40%, *P* < 0.0001 for others), indicating some effect of reduced nutrition. However, for 20% and 40%, the equivalent PL and PBS concentrations were also significantly different from each other (*P* < 0.0001), suggesting that the effect of PL on growth rate could not be entirely due to changes in nutrition. There was no significant difference in lag time between any of the treatments (Figure 6C), indicating that PL does not affect the onset of exponential growth.

## Discussion

Defining the potential of PL to inhibit bacterial growth is relevant because of the substantial innovation gap in the field of antibiotic discovery and the concerning development of pathogens that are increasingly resistant to antibiotics. The overarching hypothesis underpinning the experiments presented here was that PL displays antibacterial effects. Encouragingly, our results show that equine PL does not support bacterial growth *in vitro* and instead exhibits growth-inhibiting capacity against gram-positive and gram-negative bacteria.

We began our investigation by designing a straightforward end-point *in vitro* assay, the rationale for which was twofold. First, we were concerned that PL as a blood-derived product rich in growth factors and other bioactive serum proteins would support rather than inhibit bacterial growth (17). Second, we sought to initially evaluate PL at 100% concentration because this is the strength at which it would likely be used for any possible clinical applications. This initial set of experiments encouragingly resulted in a significant reduction in bacterial proliferation, and suggested that bacterial growth could not occur in the presence of 100% PL. We then based our subsequent analysis on a time-course growth curve assay that enabled us to monitor and quantify bacterial content more precisely over time, and to compare the effect of different PL concentrations. Even though each of the bacteria tested showed unique growth patterns, all strains exhibited a slower growth rate and lower bacterial yield in the presence of PL. More specifically, there appeared to be a delay in the onset of exponential growth for *E. coli* and *P. aeruginosa* in the presence of PL, as indicated by an increase in the length of the lag phase. We could not determine if this effect was due to a delay in the onset of growth of all bacteria in culture, or if there was an immediate, but partial, bactericidal effect, followed by the growth of the surviving bacteria. We acknowledge this as a limitation of the growth curve assay, which provides valuable information about bacterial growth dynamics but does not differentiate between bactericidal and bacteriostatic effects. Published data would suggest that platelet-derived products generally exhibit a strong antimicrobial effect characterized by an initial transient bactericidal effect, without complete elimination fully signifying an overall bacteriostatic effect (18–22). This is relevant to future clinical applications of PL as it could inform the development of a dosing regimen: a single application may have a limited effect, but administering fresh PL at repeated follow-up time intervals could be more effective at eliminating all bacteria. This will be investigated in future studies. Importantly, if PL is eventually used in a clinical setting to treat infections, it will likely be used at 100% concentration (i.e., undiluted). However, the fact that inhibitory effects on bacterial growth were observed with concentrations below 100% (in fact, for all strains 20% PL caused a significant reduction in bacterial yield and growth rate) means that PL has the potential to be an effective antimicrobial treatment.

A survey of the literature reveals some disparity in the antimicrobial effects of human platelet-derived products. An “extract liquid” obtained from activation and centrifugation of PRP was found to have a longer-lasting effect against *S. aureus* than activated PRP over 24 h (23). In other studies, activated PRP was shown to be effective against bacteria that are known to cause wound infections such as *S. aureus* and *E. coli* (20, 24, 25) but not against *K. pneumonia, E. faecalis*, or *P. aeruginosa* (24, 26). Two independent studies showed that activated PRP had antibacterial effects against *S. aureus*, but not *E. coli* or *Pseudomonas* in human dermal ulcers (27) and in a rabbit spinal infection model (28). A reduced effect of platelet-derived products on *P. aeruginosa* has been reported in other studies (21, 29), and some have even suggested that growth may be enhanced (24, 25). In contrast, in the present study, PL inhibited the growth of all four strains tested including *P. aeruginosa*, with no evidence of regrowth or growth stimulation. Differential susceptibility has been reported for other platelet-derived biologicals (30) and is thought to be due to phenotypic differences between bacterial strains, such as cell membrane charge and composition. In the present study we found that PL inhibited the growth of the gram-negative bacteria in a concentration-dependent manner, whereas the growth of gram-positive bacteria was similarly affected regardless of PL concentration. This finding may be an indication of bacterial sensitivity to the active components in PL and the mechanism by which antimicrobial peptides interact with the surface proteins of different bacteria. Even though we acknowledge that only four bacterial strains were included in the current study, this correlation opens the opportunity to perform future studies on the potential strain-specific antimicrobial effects of PL.

A limitation of the *in vitro* growth curve assay is the potential effect of nutrient deprivation on bacterial growth. The assay utilizes a liquid culture, meaning that treating bacteria with 100% PL does not allow for the addition of BHI growth medium. This would in turn reduce the supply of nutrients, which could confound the direct effect of PL on bacterial growth. To overcome this, we tested different concentrations of PL (in BHI) and matched them with a parallel set of PBS dilutions. As expected, our results show that reducing BHI had a direct effect on bacterial growth dynamics, however, a reduction in maximum bacterial yield could not be measured until BHI content was reduced to 40% (i.e., 60% PL or PBS concentration), indicating that any effects on growth at lower concentrations are likely due to presence of PL and not lack of BHI. For *E. coli*, the maximum bacterial yield was significantly different between all concentrations of PL vs. PBS except 100%, and for *S. aureus* and *P. aeruginosa* this was true up to 60% PL compared to PBS.

In our initial *in vitro* end-point assay, we consistently observed the formation of a bacterial aggregate when 100% PL was added to a *S. aureus* culture. This floating biomass, also termed a biofloat, has been reported in synovial fluid (31, 32), and similar structures have been observed in chronic wound infections (33). Much like the more typical adherent biofilm, this aggregation protects the bacteria from host defense mechanisms and antibiotics, thus increasing the severity of infection and making it more challenging to treat (34) *S. aureus* aggregation has also been noted to occur in serum byproducts as a result of an interaction between surface receptors and fibrinogen (35, 36). We are aware that PL contains clotting factors including fibrinogen, and therefore a similar mechanism may be responsible for biofloat formation following PL exposure to *S. aureus*. Bacterial aggregation did not occur in the growth curve assay, most likely due to differences in the experimental set up such as a smaller volume, use of flat-bottom wells rather than conical tubes, and constant agitation. The interaction between *S. aureus* and PL clearly has major implications regarding the application of PL as an antimicrobial alternative for this particular bacterium and we are currently pursuing specific PL modifications that may counteract biofloat formation and increase its efficacy as an antimicrobial alternative.

In conclusion, our data demonstrate that equine PL exhibits significant antimicrobial activity against bacterial strains commonly found in orthopedic and postoperative infections. Our findings highlight the potential value of PL as a broad-spectrum antimicrobial deserving further, more detailed analysis. This, together with its known anti-inflammatory effects, Naskou et al. (37) could define PL as a multi-functional treatment, helping to control both inflammation and infection to promote the healing process.

## Data Availability Statement

The raw data supporting the conclusions of this article will be made available by the authors, without undue reservation.

## Author Contributions

The project was conceived by JG, SÁ-N, and JP. All experiments and data analysis were performed by JG and SÁ-N. JG and JP are responsible for manuscript preparation. All authors contributed to the article and approved the submitted version.

## Conflict of Interest

The authors declare that the research was conducted in the absence of any commercial or financial relationships that could be construed as a potential conflict of interest.

## Publisher's Note

All claims expressed in this article are solely those of the authors and do not necessarily represent those of their affiliated organizations, or those of the publisher, the editors and the reviewers. Any product that may be evaluated in this article, or claim that may be made by its manufacturer, is not guaranteed or endorsed by the publisher.
