# Supplementary figures and images for: Antimicrobial Effects of Equine Platelet Lysate
Source: Front Vet Sci. 2021 Aug 19;8:703414. doi: 10.3389/fvets.2021.703414 (PMC8416987; doi:10.3389/fvets.2021.703414)

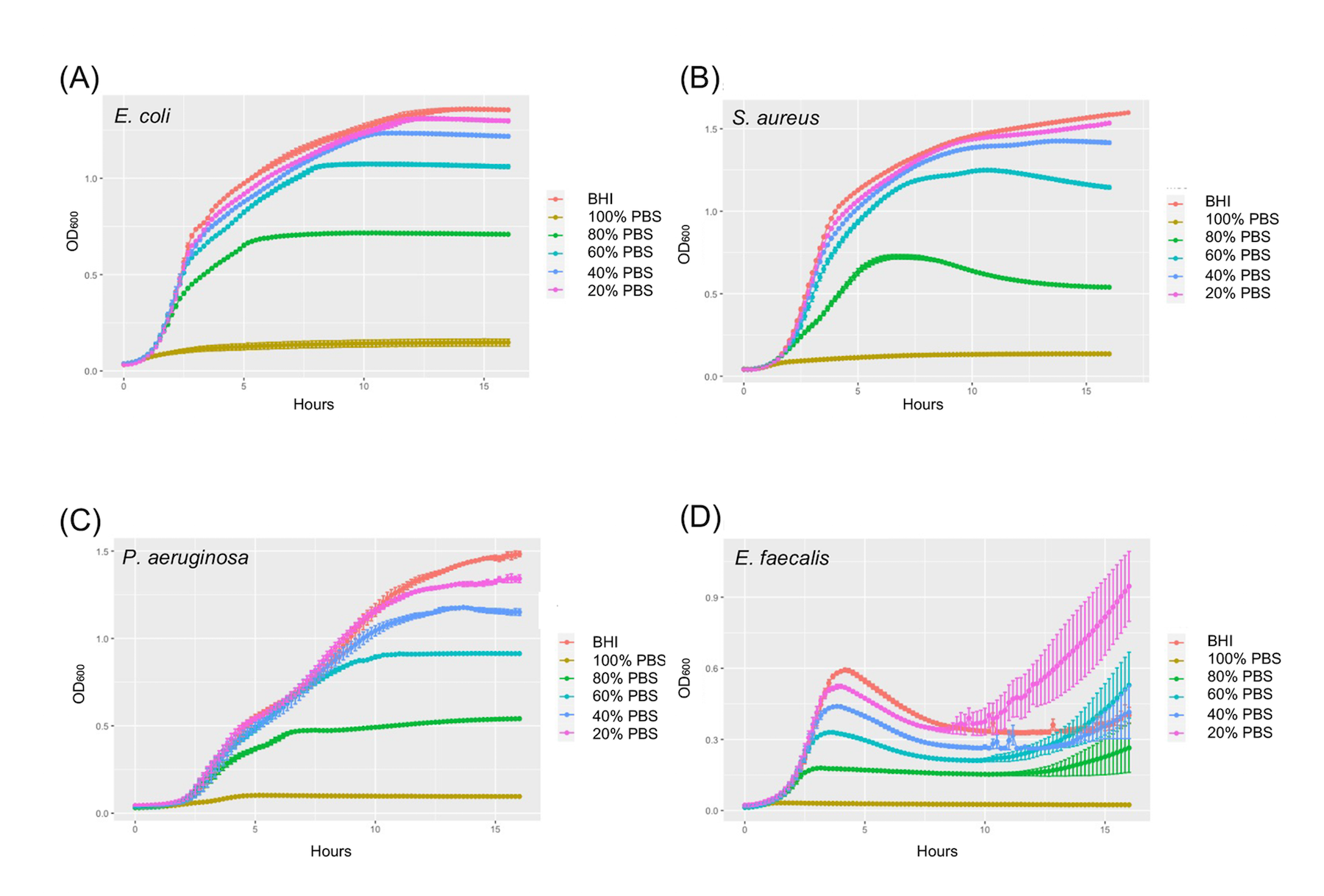

Supplement: Supplementary file 1 [file Image_1.tif]
